# Nasal and tracheobronchial nitric oxide production and its influence on oxygenation in horses undergoing total intravenous anaesthesia

**DOI:** 10.1186/s12917-022-03234-3

**Published:** 2022-04-11

**Authors:** Henriette L. Wilkens, Stephan Neudeck, Sabine B. R. Kästner

**Affiliations:** 1 Clinic for Horses, University of Veterinary Medicine Hannover, Foundation, Hannover, Germany; 2 Clinic for Small Animals, University of Veterinary Medicine Hannover, Foundation, Hannover, Germany

**Keywords:** Horses, Anaesthesia, Nitric oxide, Hypoxaemia, Endotracheal intubation

## Abstract

**Background:**

The present study aimed to investigate the effect of endotracheal intubation on nasal and tracheal endogenous NO concentrations, gas exchange and oxygenation in horses undergoing general anaesthesia. In many species a major part of physiological nitric oxide (NO) production takes place in the nasopharynx. Inhaled NO acts as a pulmonary vasodilator and regulates lung perfusion and endotracheal intubation bypasses the nasopharynx. Six horses were randomly assigned to either the “intubated” (INT) or the “non-intubated” (nINT) treatment group. Horses were premedicated with dexmedetomidine (5 μg/kg IV). Anaesthesia was induced with 2.5 mg/kg ketamine and 0.05 mg/kg diazepam IV, and it was maintained by administration of a triple-drip (100 mg/kg/h guaifenesin, 4 mg/kg/h ketamine, 7 μg/kg/h dexmedetomidine). The horses were spontaneously breathing room air. Heart rate, cardiac output, arterial blood pressure, pulmonary arterial blood pressures and respiratory rate were recorded during a 100-min anaesthesia period. Arterial, venous and mixed venous blood samples were taken every 10 minutes and analysed for partial pressure of oxygen (PO_2_) and carbon dioxide (PCO_2_), oxygen saturation and haemoglobin content. Standard oxygenation indices were calculated. Nasal and tracheal endogenous NO concentration was determined by chemiluminescence.

**Results:**

Cardiovascular variables, respiratory rate, PO_2_, PCO_2_, oxygen saturation, haemoglobin content, CaO_2_, O_2_ER, P_(a-ET)_CO_2_ and Q_s_/Q_t_ did not differ significantly between the two treatment groups. The P_(A-a)_O_2_ was significantly higher in INT (6.1 ± 0.3 kPa) compared to nINT (4.9 ± 0.1 kPa) (*p* = 0.045), respectively. The nasal (8.0 ± 6.2 ppb) and tracheal (13.0 ± 6.3 ppb) endogenous NO concentration differed significantly in INT (*p* = 0.036), but not in nINT (nasal: 16.9 ± 9.0 ppb; tracheal: 18.5 ± 9.5 ppb) (*p* = 0.215).

**Conclusion:**

Endotracheal intubation reduces the nasal and tracheal endogenous NO concentration. The influence on pulmonary gas exchange and oxygenation is negligible in horses breathing room air.

## Background

Hypoxaemia is considered to be a potential risk factor for the high perioperative mortality and morbidity in horses. However, strong scientific evidence is still lacking [1, 2]. Severe hypoxaemia in horses has been associated with significant complications such as cardiac arrest [3], post anaesthetic cerebral necrosis [4], post anaesthetic myositis [5] and wound infection [6]. The main aetiologies of hypoxaemia during anaesthesia include a ventilation and perfusion mismatch and an increased shunt fraction of blood (Q_s_/Q_t_) due to atelectasis [7, 8]. A variety of techniques have been developed to prevent and reduce the development of hypoxaemia. Besides various ventilation strategies [9] and the administration of aerosolised beta agonists [10], the inhalation of exogenous nitric oxide (NO) is an effective approach in treating hypoxaemia [11]. Endogenous NO plays an important role in the regulation of vascular and bronchial tone and is produced by the enzyme NO-synthase in the endothelial cells of various tissues [12, 13]. The pharmacodynamic effect of inhaled NO at low concentrations is limited to the pulmonary vessels, where it serves as a potent vasodilator in adequately ventilated alveoli, leading to improved gas exchange due to an optimized ventilation-perfusion ratio (V_A_/Q) [14, 15]. In humans and various animal species, a large part of the physiological endogenous NO production in the respiratory tract originates from the nasopharynx [16, 17]. In endotracheally intubated human subjects, the endogenous NO concentration in the lungs is lower than in non-intubated subjects, due to the inhaled air bypassing the nasopharynx [18]. In horses, the main origin of bronchial NO has not conclusively been defined. The horse as an obligatory nasal breather may benefit from endogenous NO synthesis in the nasopharynx as this may improve the pulmonary V_A_/Q ratio. In this case, conventional endotracheal intubation in the horse during general anaesthesia could prevent the physiological aerogenic transport of endogenous NO and its effect on pulmonary perfusion. This could result in deterioration of gas exchange and oxygenation. We hypothesised that circumvention of the nasal passages by endotracheal intubation will impair gas exchange and oxygenation in horses undergoing total intravenous anaesthesia and breathing room air. Therefore, the aim of the study was to investigate the effect of endotracheal intubation of horses on nasal and bronchial endogenous NO concentrations, on pulmonary gas exchange and on oxygenation indices during triple drip anaesthesia compared to non-intubated horses.

## Results

### Anaesthesia

After the initial bolus of dexmedetomidine, all horses showed moderate to good sedation. In INT, all horses required additional sedation before induction of anaesthesia. In nINT, three of six horses needed further dexmedetomidine.

In both treatment groups, 60.0% (INT) and 66.7% (nINT) of the horses displayed insufficient depth of anaesthesia after reduction of triple-drip which was marked by swallowing, nystagmus, movement of the ears, snorting and neighing without any surgical stimulus, which required an increase in the infusion rate of the triple-drip. During triple-drip, group INT required 3.1 ± 0.6 mg/kg/h ketamine, 5.4 ± 1.0 μg/kg/h dexmedetomidine and 77.9 ± 15.1 mg/kg/h guaifenesin to ensure adequate general anaesthesia. In nINT, horses received 3.0 ± 0.5 mg/kg/h ketamine, 5.3 ± 1.0 μg/kg/h dexmedetomidine and 75.9 ± 13.6 mg/kg/h guaifenesin. The non-intubated horses developed an obvious nasal stridor soon after induction of anaesthesia.

### Recovery

The recovery was calm and controlled in all horses. It took the horses 36.2 ± 11.7 min in group INT and 36.2 ± 8.6 min in group nINT, respectively, to regain a secure standing position (*p* = 0.813). The median recovery score was 17.5 (13–26) and 15.5 (11–26) in the INT and nINT group, respectively (*p* = 0.125).

### Cardiopulmonary data

Cardiovascular and pulmonary data did not differ significantly between INT and nINT (Table 1).

**Table 1:** Cardiovascular and respiratory data and calculations during general anaesthesia of six horses investigated in the “intubated” (INT) and “non-intubated” (nINT) treatment groups

		Time of measurement												
Parameter	Treatment group (***n*** = 6)	Baseline (B)	Sedation (S)	A10	A20	A30	A40	A50	A60	A70	A80	A90	A100	*p*-value (group comparison)
HR (beats/minute)	INT			36.0 ± 5.0	34.0 ± 4.0	34.0 ± 2.0	34.0 ± 2.0	35.0 ± 4.0	36.0 ± 4.0	38.0 ± 4.0	35.0 ± 1.0	34.0 ± 2.0	35.0 ± 2.0	0.538
	nINT			35.0 ± 2.0	35.0 ± 2.0	35.0 ± 3.0	36.0 ± 3.0	37.0 ± 4.0	38.0 ± 4.0	38.0 ± 4.0	36.0 ± 3.0	35.0 ± 4.0	35.0 ± 5.0	
MAP (mmHg)	INT			112 ± 5	107 ± 17	112 ± 14	117 ± 42	98 ± 3	99 ± 13	98 ± 10	98 ± 12	99 ± 13	97 ± 13	0.587
	nINT			115 ± 5	111 ± 8	104 ± 11	97 ± 13	96 ± 12	92 ± 12	90 ± 15	98 ± 13	99 ± 14	101 ± 16	
MPAP (mmHg)	INT	26 ± 05	29 ± 4	29 ± 5	27 ± 7	29 ± 2	29 ± 5	27 ± 5	21 ± 1	24 ± 4	24 ± 2	27 ± 5	29 ± 8	0.311
	nINT	26 ± 5	36 ± 4	27 ± 7	26 ± 7	24 ± 11	23 ± 10	22 ± 11	21 ± 10	20 ± 13	21 ± 10	23 ± 3	23 ± 3	
CI (mL/min/kg)	INT					85.8 ± 22.6		85.0 ± 12.1		88.7 ± 16.1		83.8 ± 15.9		0.375
	nINT					72.2 ± 4.9		85.7 ± 9.3		85.7 ± 7.7		80.9 ± 15.0		
SVI (mL/kg)	INT					2.5 ± 0.7		2.4 ± 0.4		2.4 ± 0.6		2.5 ± 0.5		0.253
	nINT					2.0 ± 0.2		2.3 ± 0.2		2.3 ± 0.2		2.3 ± 0.6		
SVRI (dynes/s/cm)	INT					110.5 ± 32.4		94.1 ± 17.4		90.3 ± 15.4		96.1 ± 15.7		0.953
	nINT					114.7 ± 15.3		89.8 ± 8.2		84.8 ± 15.7		100.0 ± 17.1		
RR (breaths/minute)	INT			14.0 ± 2.0	13.0 ± 3.0	17.0 ± 3.0	16.0 ± 5.0	16.0 ± 4.0	17.0 ± 4.0	17.0 ± 7.0	19.0 ± 9.0	17.0 ± 6.0	15.0 ± 5.0	0.667
	nINT			14.0 ± 3.0	14.0 ± 5.0	14.0 ± 8.0	15.0 ± 6.0	15.0 ± 5.0	15.0 ± 5.0	16.2 ± 6.0	16.0 ± 6.0	14.0 ± 5.0	15.0 ± 4.0	
FiO_2_	INT	0.2	0.2	0.2	0.2	0.2	0.2	0.2	0.2	0.2	0.2	0.2	0.2	
	nINT	0.2	0.2	0.2	0.2	0.2	0.2	0.2	0.2	0.2	0.2	0.2	0.2	
PaO_2_ (kPa)	INT	13.8 ± 3.4	11.0 ± 1.2	7.1 ± 0.7^b^	6.9 ± 0.7^b^	6.7 ± 1.1^b^	5.9 ± 0.9^b^	5.9 ± 0.7^b^	5.7 ± 0.8^b^	5.5 ± 1.1^b^	5.7 ± 1.0^b^	5.9 ± 0.6^b^	5.8 ± 0.9^b^	0.613
	nINT	13.2 ± 1.2	11.4 ± 1.7	6.8 ± 0.9^b^	6.4 ± 0.7^b^	6.3 ± 0.7^b^	6.1 ± 0.6^b^	6.2 ± 0.6^b^	5.9 ± 0.4^b^	6.2 ± 0.5 ^b^	6.3 ± 0.5^b^	6.4 ± 0.3^b^	6.4 ± 0.7^b^	
PaCO_2_ (kPa)	INT	5.4 ± 0.4	5.7 ± 0.4^b^	5.9 ± 0.8	6.1 ± 0.5	5.6 ± 0.6	5.9 ± 0.3	5.8 ± 0.3	5.5 ± 0.4	5.7 ± 0.3	5.8 ± 0.4	5.7 ± 0.2	5.8 ± 0.7	0.072
	nINT	5.4 ± 0.3	5.7 ± 0.3	6.1 ± 0.5	6.6 ± 0.5^b^	6.3 ± 0.8	6.1 ± 0.7	6.3 ± 0.8^b^	6.3 ± 0.7^b^	6.2 ± 0.7	6.3 ± 0.7	6.4 ± 0.8	6.5 ± 0.8^b^	
SaO_2_ (%)	INT	95.7 ± 1.2	95.7 ± 2.4	90.0 ± 2.4^b^	89.5 ± 3.2^b^	88.9 ± 3.4^b^	85.9 ± 3.2^b^	85.6 ± 3.5^b^	86.4 ± 4.2^b^	82.8 ± 5.0^b^	84.3 ± 6.3^b^	85.7 ± 3.3^b^	84.0 ± 6.2^b^	0.686
	nINT	96.1 ± 0.3	95.8 ± 1.0	89.3 ± 2.9^b^	86.8 ± 2.6^b^	87.2 ± 3.8^b^	86.6 ± 3.1^b^	86.4 ± 2.9^b^	85.3 ± 2.3^b^	86.7 ± 2.5^b^	87.2 ± 1.9^b^	86.5 ± 3.1^b^	87.4 ± 3.4^b^	
Hb (g/dL)	INT	16.0 ± 2.8	12.5 ± 0.8	12.1 ± 1.4	11.6 ± 0.7	11.0 ± 0.9^b^	10.3 ± 1.2^b^	10.0 ± 1.3^b^	11.3 ± 2.2^b^	10.5 ± 1.8	10.2 ± 1.4^b^	9.7 ± 1.0^b^	10.4 ± 1.0^b^	0.199
	nINT	14.7 ± 1.9	11.5 ± 0.9^b^	11.6 ± 1.3 ^b^	10.7 ± 0.5^b^	10.1 ± 1.4^b^	9.7 ± 1.2^b^	9.6 ± 1.3^b^	9.3 ± 1.2^b^	9.3 ± 1.3^b^	9.5 ± 1.4^b^	9.3 ± 1.2^b^	10.0 ± 2.3^b^	
CaO_2_ (mL/L)	INT			14.7 ± 1.6	14.1 ± 0.6	13.2 ± 1.1	12.0 ± 1.5	11.6 ± 1.6	13.1 ± 2.4	11.8 ± 2.4	11.7 ± 2.3	11.2 ± 1.2	11.9 ± 1.4	0.309
	nINT			14.0 ± 1.3	12.5 ± 0.8	12.0 ± 2.0	11.4 ± 1.8	11.3 ± 1.8	10.8 ± 1.5	10.9 ± 1.8	11.2 ± 1.8	10.9 ± 1.4	11.9 ± 3.0	
PAO_2_ (kPa)^a^	INT			11.8 ± 1.0	11.4 ± 0.6	12.2 ± 1.1	12.1 ± 0.6	12.2 ± 0.8	12.6 ± 1.1	12.5 ± 0.6	12.6 ± 0.5^b^	12.0 ± 0.3	12.5 ± 0.9	0.024^a^
	nINT			11.3 ± 0.6	10.7 ± 0.7	11.1 ± 1.1	11.3 ± 0.9	11.2 ± 0.8	11.2 ± 0.6	11.5 ± 0.8	11.3 ± 0.6	11.4 ± 1.2	11.3 ± 1.4	
P_(A-a)_O_2_ (kPa)^a^	INT			4.7 ± 1.0	4.5 ± 0.9	5.6 ± 1.7	6.1 ± 1.3	6.3 ± 1.3	6.7 ± 1.5	5.4 ± 0.2	6.8 ± 0.7^b^	6.1 ± 0.4	6.7 ± 0.9	0.045^a^
	nINT			4.5 ± 0.9	4.4 ± 0.7	4.8 ± 1.1	5.2 ± 1.0	5.0 ± 0.9	5.3 ± 0.7	5.4 ± 0.7	5.0 ± 0.7	5.0 ± 1.1	4.8 ± 0.9	
P_(a-ET)_CO_2_ (kPa)	INT			1.7 ± 1.0	2.2 ± 1.6	1.0 ± 0.2	1.6 ± 1.3	1.7 ± 0.9	1.6 ± 0.8	1.6 ± 0.7	1.8 ± 1.0	2.2 ± 1.4	1.8 ± 0.9	0.148
	nINT			1.0 ± 0.7	1.2 ± 0.6	1.3 ± 1.1	1.2 ± 0.5	1.0 ± 0.5	1.5 ± 0.8	1.6 ± 1.2	1.4 ± 1.0	2.4 ± 1.8	1.2 ± 1.1	
DO_2_I (L/kg/min)	INT					11.3 ± 3.3		9.8 ± 1.4		10.7 ± 3.8		9.5 ± 2.7		0.293
	nINT					8.7 ± 1.9		9.6 ± 1.7		9.3 ± 1.2		8.7 ± 1.1		
O_2_ER (%)	INT					32.0 ± 16.3		30.2 ± 6.1		30.9 ± 10.6		30.0 ± 10.0		0.177
	nINT					34.1 ± 8.5		35.0 ± 6.4		36.5 ± 5.5		38.6 ± 10.5		
Q_s_/Q_t_ (%)	INT			21.6 ± 5.0	23.3 ± 6.1	29.6 ± 6.5	42.2 ± 22.8	35.6 ± 7.4	37.6 ± 15.0	41.5 ± 13.1	36.7 ± 16.1	36.7 ± 10.1	38.3 ± 16.4	0.075
	nINT			23.5 ± 8.5	31.2 ± 11.2	30.2 ± 8.0	30.2 ± 10.3	30.8 ± 5.1	30.7 ± 4.0	29.3 ± 1.6	28.2 ± 4.9	29.3 ± 7.5	27.0 ± 10.1	

*HR* Heart rate, *MAP* Mean arterial blood pressure, *MPAP* Mean pulmonary arterial blood pressure, *CI* Cardiac index, *SVI* Stroke volume index, *SVRI* Systemic vascular resistance, *RR* Respiratory rate, *F*_*i*_*O*_*2*_ Fraction of inspired oxygen, *E*_*T*_*CO*_*2*_ End-tidal carbon dioxide, *PaO*_*2*_ Oxygen partial pressure, *PaCO*_*2*_ Arterial carbon dioxide tension, *SaO*_*2*_ Arterial oxygen saturation, *Hb* Haemoglobin, *CaO*_*2*_ Arterial oxygen content, *PAO*_*2*_ Alveolar oxygen partial pressure, *P*_*(A-a)*_*O*_*2*_ Alveolar-arterial oxygen difference, *P*_*(a-ET)*_*CO*_*2*_ Arterial-end-tidal carbon dioxide pressure difference, *DO*_*2*_*I* Oxygen delivery, *O*_*2*_*ER* Oxygen extraction ratio, *Q*_*s*_*/Q*_*t*_ Shunt fraction

^a^Significantly different compared to nINT

^b^Significantly different from baseline value (B)

### Blood gas values and calculated parameters

Under general anaesthesia, all horses were markedly hypoxaemic. In the intubated horses the mean P_a_O_2_ during the observation time was 6.1 ± 0.2 kPa and 6.3 ± 0.2 kPa in non-intubated horses. Blood gas analysis and the derived oxygenation variables did not differ significantly between the treatment groups, except for P_A_O_2_ and P_(A-a)_O_2_. The P_A_O_2_ was 12.2 ± 0.3 kPa and 11.2 ± 0.3 kPa in INT and nINT, respectively (*p* = 0.025). The P_(A-a)_O_2_ was larger in INT (6.1 ± 0.3 kPa) than in nINT (4.9 ± 0.2 kPa) (*p* = 0.045). The complete data set is presented in Table 1.

### Nitric oxide

**Fig. 1:**
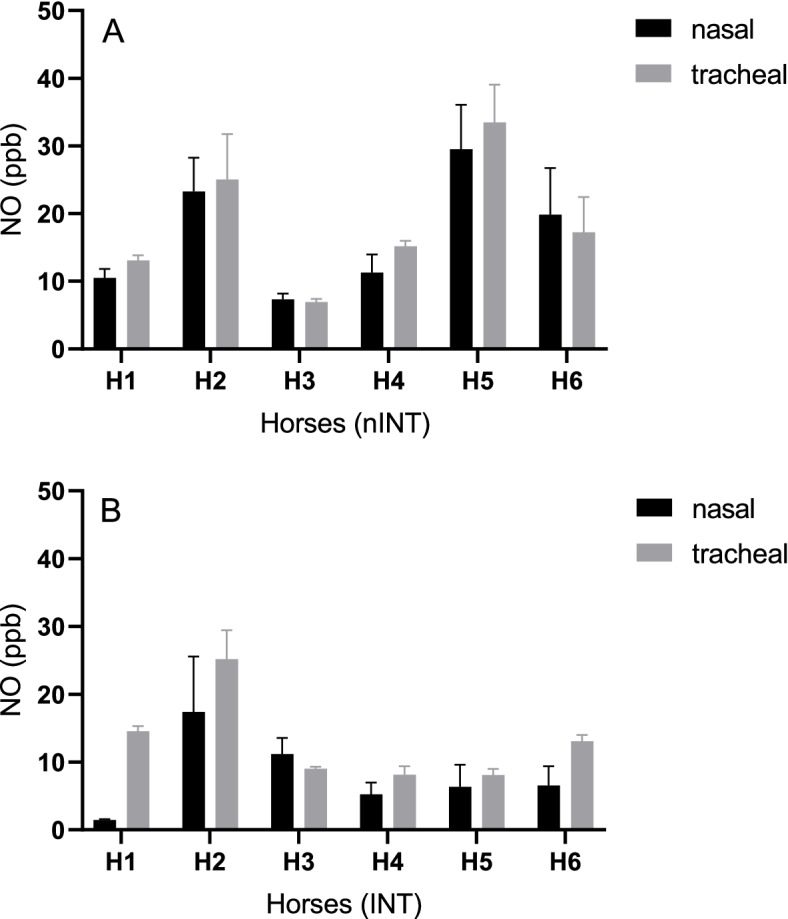
**a** Bar graph, mean nasal and tracheal nitric oxide (NO) concentrations (ppb) with standard deviation measured by chemiluminescence in six horses (H1-H6) of the “non-intubated” treatment group (nINT) under total intravenous anaesthesia. **b** Bar graph, mean nasal and tracheal nitric oxide (NO) concentrations (ppb) with standard deviation measured by chemiluminescence in six horses (H1-H6) of the “intubated” treatment group (INT) under total intravenous anaesthesia

**Fig. 2:**
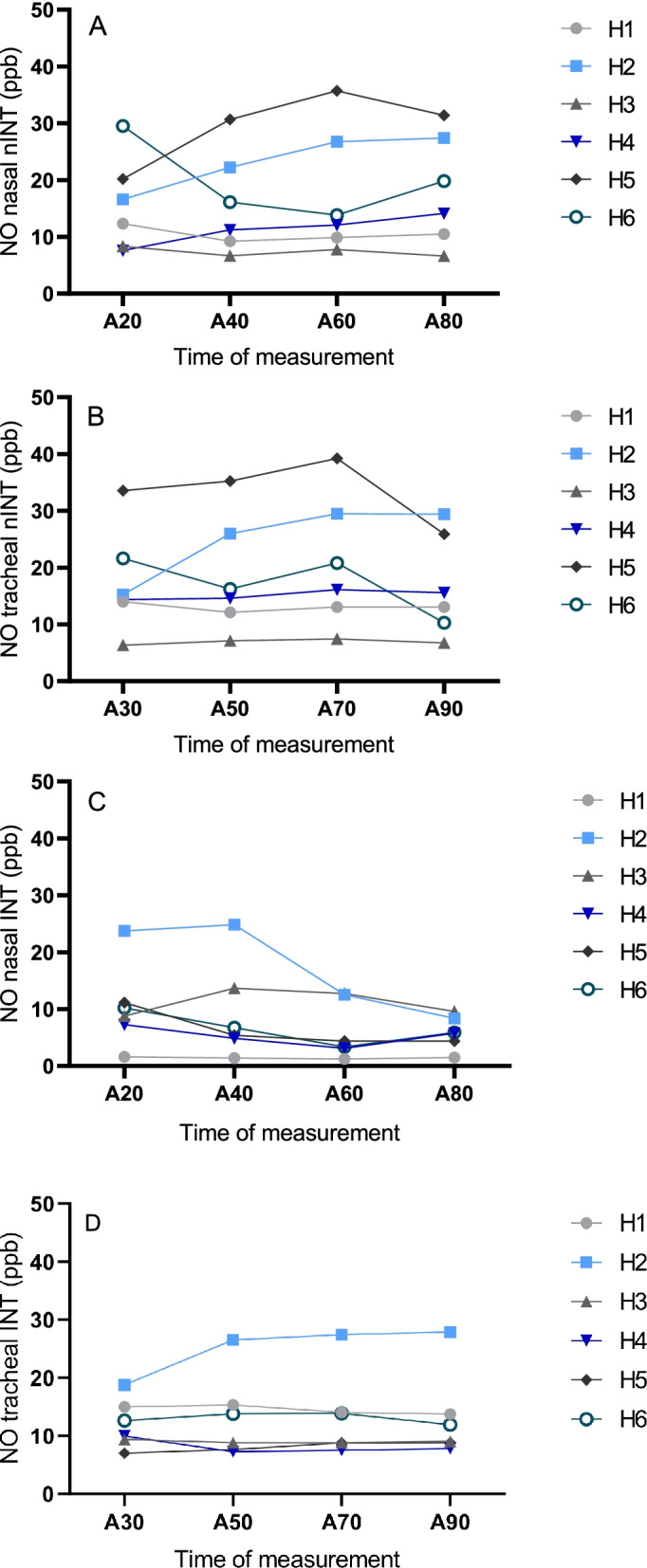
**a** Line graph, individual development of nasal nitric oxide (NO) concentrations of six horses (H1-H6) of the “non-intubated” treatment group (nINT) measured by chemiluminescence under total intravenous anaesthesia at time of measurement A20, A40, A60 and A80. **b** Line graph, individual development of tracheal nitric oxide (NO) concentrations of six horses (H1-H6) of the “non-intubated” treatment group (nINT) measured by chemiluminescence under total intravenous anaesthesia at time of measurement A30, A50, A70 and A90. **c** Line graph, individual development of nasal nitric oxide (NO) concentrations of six horses (H1-H6) of the “intubated” treatment group (INT) measured by chemiluminescence under total intravenous anaesthesia at time of measurement A20, A40, A60 and A80. **d** Line graph, individual development of tracheal nitric oxide (NO) concentrations of six horses (H1-H6) of the “intubated” treatment group (INT) measured by chemiluminescence under total intravenous anaesthesia at time of measurement A30, A50, A70 and A90

Under general anaesthesia, the nasal (8.0 ± 6.2 ppb) and tracheal (13.0 ± 6.3 ppb) endogenous NO concentration differed significantly in INT (*p* = 0.036), but not in nINT (nasal: 16.9 ± 9.0 ppb; tracheal: 18.5 ± 9.5 ppb) (*p* = 0.215). Figures 1 and 2 represent the different endogenous NO concentrations in INT and nINT during anaesthesia. The levels of endogenous NO (trending basis) varied greatly between horses (Fig. 2a-c). Overall, the non-intubated horses had higher nasal and tracheal endogenous NO concentrations compared to the intubated horses. The nasal endogenous NO concentration of non-intubated horses was 52.7% higher than in intubated horses (*p* = 0.059). The tracheal endogenous NO concentration in non-intubated horses was 29.7% higher than in intubated horses (*p* = 0.269). Both groups presented higher endogenous NO concentrations in the trachea than in the nasopharynx.

## Discussion

This study indicates that in non-intubated horses the nasal and tracheal endogenous NO concentrations were higher than in intubated horses. However, the overall effect on gas exchange and oxygenation was minimal and hypoxaemia developed quickly in horses of both treatment groups in lateral recumbency under room air respiration.

**Fig. 3:**
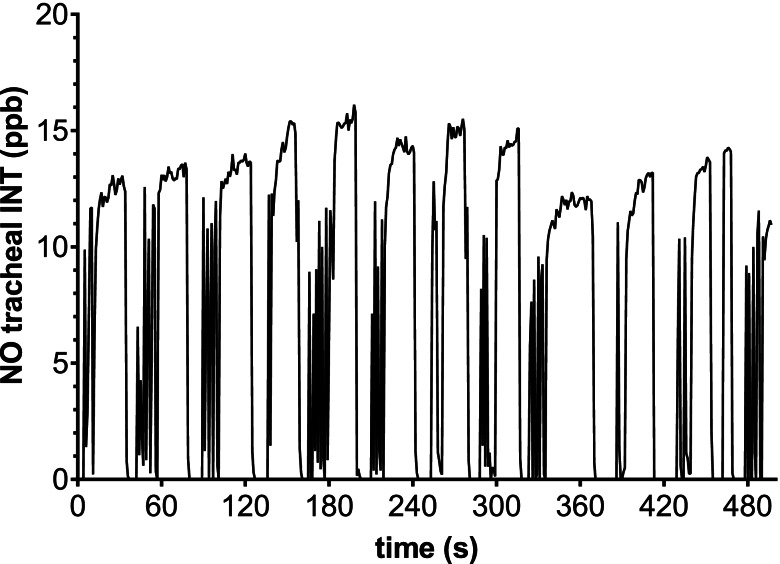
Exemplary illustration of inhalation and exhalation-related fluctuations within the NO measurement for horse 1 of group INT (measurement A30)

In various species, endogenous NO is present in large quantities in the upper respiratory tract [16, 19]. In horses, the main origin of physiologic endogenous NO in the respiratory tract has not yet been defined. In a pre-trial, we were not able to detect an endogenous NO signal in the respired gas of healthy, standing, unsedated and sedated horses. However, during TIVA we could successfully demonstrate nasal and tracheal endogenous NO in non-intubated and intubated horses. The lower limit of quantification of NO by the chemiluminescence analyser used can be as low as was 0.06 ppb. It has been shown in humans that the detectable NO concentration depends on the respiratory flow rate [20]. The lack of a detectable NO signal in the standing horses could therefore, be related to the higher respiratory flow and the altered flow conditions in standing horses compared to anaesthetized horses. The graphical analysis of the course of our measured endogenous NO concentrations in connection with the results of previous studies in other animal species [17] allows the assumption that endogenous NO concentrations were higher in the exhalation phase and the expiratory pause during no flow conditions (Fig. 3).

The presence of nasal endogenous NO in intubated horses in general anaesthesia indicates that NO production may also occur in the nasopharynx of horses. The carry-over of NO from bronchial production into nasal readings can be excluded, as endotracheal intubation separates the lungs and trachea from the ventral nasal passage and paranasal sinuses. In humans, breath-holding results in an increase in endogenous NO concentration, as a result of continued endogenous NO synthesis in the absence of respiratory flow [21]. In addition, it has been demonstrated that endotracheal intubation reduces the concentration of endogenous NO in the lungs and leads to accumulation of endogenous NO in the nasopharynx [18]. In the current study, we observed a plateau phase instead of an accumulation of endogenous NO in the nasopharynx. The reason for this difference remains unclear. The lack of respiratory flow across the nasal mucosa can reduce the stimulus to release of endogenous NO into stagnant air [22]. The presence of nasal airflow with concurrent stimulation of endogenous NO release and possible carry-over from bronchial NO in non-intubated horses could explain the about 50% higher nasal endogenous NO concentration compared to intubated horses [22]. There was also an individual variation in the detected endogenous NO levels which might be related to variations in the breathing pattern and respiratory flow rates contributing to differences in dilution factors [23].

Bronchial endogenous NO production plays an important role in the horse as shown by the 38.5 and 8.6% higher bronchial endogenous NO compared to nasal endogenous NO concentrations in INT and nINT, respectively. The pulmonary tissue itself contributes significantly to the NO measured in the bronchial tree as demonstrated by the high bronchial endogenous NO levels in the lungs of intubated horses. The high bronchial endogenous NO concentration in both groups are possibly related to increased bronchial endogenous NO synthesis due to increased hypoxic stress on the lungs during general anaesthesia [24]. In the standing horses a bronchial endogenous NO signal could not be detected. The comparison of patients with overt hypoxaemia and those with normoxaemia or hyperoxaemia could potentially elucidate the aetiology of NO release in horses during general anaesthesia.

Even though overall bronchial endogenous NO concentrations were higher in non-intubated horses than in intubated horses, both groups developed moderate hypoxaemia as indicated by low PaO_2_; low SaO_2_ and low CaO_2_immediately after induction of anaesthesia and recumbency without further progression over time. This emphasizes that the induction of general anaesthesia in healthy horses and their subsequent positioning in lateral recumbency under room air respiration is sufficient to induce moderate hypoxaemia in a rapid manor [2]. The main aetiologies of hypoxaemia related to positioning during anaesthesia include alterations of oxygenation such as ventilation and perfusion mismatch and an increased Q_s_/Q_t_due to compression atelectasis [7, 8].

The guaifenesin-dexmedetomidine-ketamine TIVA protocol used in this study resulted in a very superficial stage of anaesthesia as demonstrated by the occurrence of spontaneous movement without any stimulation in some horses. The CI with approx. 85 ml/kg/min and mean arterial blood pressure values of 100 mmHg were in the range of awake, standing horses [25, 26]. This could be related to the superficial anaesthesia per se*,* but could also be the result of sympathetic stimulation due to hypoxaemia [27] or ketamine administration. Major overestimation of the CI by the LiDCO technique is unlikely because aberrant influence of the sensor was ruled out (see limitations).

Even though PaO_2_ is the major variable of interest in this study, SaO_2_ was derived from a human algorithm built in the blood gas machine and not by co-oximetry. Horse haemoglobin has a higher affinity for oxygen than human haemoglobin [28] and calculations based on Haymerle et al. [29] and the algorithm according to Siggaard-Andersen et al. [30], resulted in SaO_2_ values of about 5% above the measured values. An average SaO_2_ of 90 to 91%, in combination with the well preserved cardiovascular function resulting in sufficient oxygen delivery might explain the good recovery of the horses without any signs of hypoxic damage.

Occasionally, an apneustic breathing pattern was noted that was suspected to be secondary to ketamine administration. There were no signs of diffusion impairment, and F_i_O_2_ of 21% at the investigation site Hannover, Germany, 55 m above sea level results in an approximated inspired oxygen pressure of 19.6 kPa (0.21 × (100–6.3 kPa) ruling out low oxygen pressure. Formation of V_A_/Q mismatch and right to left shunt flow (Q_s_/Q_t_) by compression atelectasis are the most common causes of hypoxaemia in anaesthetized, recumbent horses [7, 8].

Development of a V_A_/Q mismatch and increase in pulmonary Q_s_/Q_t_ contribute to an increase in the P_(A-a)_O_2._ Both P_(A-a)_O_2_ and Q_s_/Q_t_ were smaller in group nINT than in group INT, but these differences did not result in an overt and clinically relevant improvement in oxygenation. Horses in nINT developed slightly higher PaCO_2_ values and thereby lower alveolar ventilation compared to horses in group INT probably as a result of partial obstruction of the upper respiratory tract, as indicated by occurrence of a nasal stridor. The higher PaCO_2_ in nINT results in lower PAO_2_ by mathematical coupling and might contribute to the difference in P_(A-a)_O_2_. In awake, healthy horses, about 1% of the cardiac output occurs as shunt flow [31]. In the current study, 20–40% of the cardiac output was not oxygenated in the lungs with a tendency for lower Q_s_/Q_t_ values in nINT. It remains speculative if this observation could be related to less atelectasis formation.

Exogenous supplementation of NO via inhalation improves oxygenation in anaesthetized horses due to better perfusion of the alveoli, which remain ventilated during anaesthesia, resulting in a reduction of P_(A-a)_O_2_ [11]. In the bronchial tree of non-intubated horses increased levels of endogenous NO could be detected but detailed analysis of dead space ventilation, V_A_/Q matching and shunt was not possible within the setup of the current study. This would have required the use of the multiple inert gas elimination technique (MIGET). It remains an unkown as to whether or not the preservation of a strong hypoxic pulmonary vasoconstrictive (HPV) response under TIVA [32] counteracted the dilatative NO effect on the pulmonary vessels and contributed to the absence of a significant relevant effect on V_A_/Q-matching. Further studies on NO effects during TIVA are needed.

Limitations include a reduced sample size, which resulted in a low study power. The pre-hoc sample size calculation was based on the assumption that a difference in PaO_2_ of 2.66 kPa would be considered a clinically relevant, justifying refraining from endotracheal intubation. Post-hoc analysis based on the observed effect size (η^2^ = 0.01455) in PaO_2_ resulted in a power of 10%, requiring 27 horses to obtain a power of 80% with an alpha of 5%.

The lithium dilution technique used in this study can be influenced by ketamine, dexmedetomidine, diazepam or guaifenesin being present in the horses’ blood [33] and CO values could be overestimated. By checking the baseline sensor voltage with contact to sodium chloride solution and to the horses’ blood before each measurement and lack of aberrant voltage values we believe that major errors can be ruled out [34].

The NO measurement by chemiluminescence is a relative measuring method and changes in pressure conditions can influence the measured values. Pressure fluctuations could be caused by a changed breathing pattern of the horse, the formation of condensation in the NO sampling lines or the aspiration of nasal mucus.

## Conclusion

Our results indicate that endotracheal intubation reduces nasal and bronchial endogenous NO concentrations in horses under general anaesthesia. Furthermore, the findings suggest that bronchial endogenous NO synthesis in horses has more impact on the overall bronchial endogenous NO content than nasal endogenous NO production. Both treatment groups developed a large P_(A-a)_O_2_ and Q_s_/Q_t_, with only minor deterioration in intubated horses with nasal bypass. The influence of endotracheal intubation on pulmonary gas exchange and oxygenation is limited in horses breathing room air. Nevertheless, the lack of endotracheal intubation in this study elicited an early clinically relevant partial obstruction of the upper airways.

## Methods

The study was designed as a prospective, randomized, experimental cross-over study. A blinding was not possible due to obvious endotracheal intubation.

### Horses

Based on a pre-hoc sample size calculation with alpha of 5%, beta of 20% and the assumption that 2.67 kPa difference in PaO_2_ would be a clinically relevant difference, the study included seven horses owned by the Clinic for Horses of the University of Veterinary Medicine Hannover, Foundation. One horse had to be removed from the study for health reasons unrelated to the study. The mean body weight of the five mares and one gelding was 552.5 ± 70.6 kg, and normally distributed. The mean age was 12.2 ± 7.8 years. Breeds included two Hanoverians, three Standardbreds and one Arabian horse. All horses were in good systemic health, based on pre-anaesthetic physical and haematologic examination. All horses went randomly through an “intubated” (INT) and a “non-intubated” (nINT) treatment arm after a drawing lot. A wash-out period between treatments was allowed for at least 10 days. The horses were kept in boxes with straw and were provided with water ad libitum and hay. In addition, the horses had daily access to a paddock and a pasture. All horses survived the trials and returned to their normal lives. All procedures were carried in accordance with the ARRIVE guidelines.

### Anaesthesia

Horses were sedated with an initial bolus of dexmedetomidine 5 μg/kg IV (Dexdomitor®, Orion Pharma, Espoo, Finland). In case of insufficient sedation an additional bolus of dexmedetomidine 2 μg/kg IV was added. General anaesthesia was induced with 2.5 mg/kg ketamine (Narketan®, Vetoquinol GmbH, Ismaning, Germany) and 0.05 mg/kg diazepam (Ziapam®, Ecuphar GmbH, Greifswald, Germany) IV. After induction, horses in the INT treatment group underwent endotracheal intubation (Cuffed endotracheal tube ET 24–30 mm, Surgivet®, Smiths Medical PM, Inc., St. Paul, USA). All horses were allowed to breathe room air spontaneously. A hoist was used to lift horses onto a padded surgical table and placed in left lateral recumbency. No top ups of ketamine or dexmedetomidine were necessary to get the horses positioned. Anaesthesia was maintained by administration of a triple-drip (100 mg/kg/h guaifenesin (Myorelax®, CP-Pharma, Burgdorf, Germany), 4 mg/kg/h ketamine, 7 μg/kg/h dexmedetomidine) using an infusion pump. After 20 min of anaesthesia, the infusion rate was halved. In case of insufficient depth of anaesthesia, the infusion rate was doubled again and supplemented with a bolus of ketamine (0.2 mg/kg). Lactated Ringer solution^f^ was infused in parallel through a y-connector at a rate of 5 mL/kg/h. For recovery, horses were hoisted into a padded recovery box. Two people on a catwalk supported the recovery phase of the horses with ropes attached to the tail and head, and the recovery quality was assessed by the main investigator using the recovery quality scale of Clark-Price et al. [35].

### Instrumentation

Prior to anaesthesia and after aseptic preparation and local anaesthesia of the skin with lidocaine 1,5 ml SC (Lidocainhydrochlorid 2%, bela-pharm GmbH & Co. KG, Vechta, Germany), a catheter (Intraflon 2 12 G, Vygon, Ecouen, France) was placed into the left jugular vein for drug administration for anaesthesia induction, sampling of venous blood and injection of lithium chloride for the cardiac output measurement. An introducer port (Exacta™, 8,5 Fr, Merit Medical, South Jordan, USA) was placed into the right jugular vein after local anaesthesia. Via this port, a custom-designed cardiac catheter (1800 mm, 7F, Gaeltec Devices Ltd., Glasgow, Scotland) was placed into the pulmonary artery verified by pressure wave forms [36] for the measurement of the pulmonary artery blood pressure (PAP) and sampling of mixed venous blood. The pulmonary catheter was pulled out prior to recovery. After induction of anaesthesia, the facial artery was canulated with a catheter (Venocan™ PLUS IV Catheter, 20 gauge, 33 mm, Kruuse A/S, Langeskov, Denmark) for measurement of the mean arterial blood pressure (MAP), cardiac output (LiDCO, London, UK) and for taking arterial blood samples for blood gas analyses. All horses were instrumented with electrocardiogram electrodes placed for base-apex lead II analysis and measuring of the heart rate. Respiratory rate, fraction of inspired oxygen (F_i_O_2_) and E_T_CO_2_ were recorded via an anaesthesia monitor (Vet.-Tech. Model JAVC 2000, J.D. Medical distributing Company, Phoenix, USA). In group INT, the gas samples for infrared capnography were taken at the Y-piece of the endotracheal tube. In group nINT, the sampling line was placed in the middle nasal passage of the horse.

### Measurements

At baseline (B) (standing, unsedated horse) and 10 min after dexmedetomidine administration (S), PAP was recorded and arterial, venous and mixed venous blood sample were taken in pre-heparinized syringes. A blood gas analyser (ABL800 FLEX, Radiometer GmbH, Laatzen, Germany) was used to determine the oxygen partial pressure (PO_2_), carbon dioxide partial pressure (PCO_2_), oxygen saturation (SO_2_) and haemoglobin content. The blood gas analyser was calibrated with a device own calibration program before each run. Within the first 10 min of anaesthesia, instrumentation was completed. After 10 min of anaesthesia and every 10 min thereafter PAP, respiratory rate, F_i_O_2_, heart rate and MAP were recorded and arterial, venous and mixed venous blood samples were collected over a period of 90 min (A10-A100). Cardiac output was attempted to be measured at the end of expiration during the spontaneous breathing of the horses after 30, 50, 70 and 90 min of anaesthesia (A30, A50, A70, A90) by use of the lithium dilution technique [37]. Before each measurement, the baseline sensor voltage in contact with the horses’ blood was compared to the sensor voltage against physiologic saline and checked for aberrant values.

Nasal and tracheal endogenous NO concentrations were measured by chemiluminescence (ANALYZER CLD88 sp., ECO Physics, Duernten, Swiss) (A20-A100). One sampling line was placed in the ventral nasal meatus at the transition to the nasopharynx and the second line was placed at the bifurcation of the trachea under endoscopic control. For NO measurements at both locations, the NO sensor was alternated between the sampling lines every 10 min. The NO measurement commenced after completion of instrumentation. Sampling flow rate was 110 ml/min. The NO concentration was measured with a data sampling rate of 1 Hz in the respired air. The NO content was expressed in parts per billion (ppb). The ANALYZER CLD88 sp. was calibrated every morning before starting the experiment with a device own calibration program. The NO concentration at a sampling site was calculated from the mean value of the maximum 10% of all values over the 10-min measurement period, to remove artefacts due to movement, pressure changes and humidity as indicated by negative or strongly fluctuating values of multifactorial origin.

### Calculated data

The following variables were calculated in accordance with standard formulae: Oxygen content in mL/L of arterial (CaO_2_) and mixed-venous blood (C ⊽ O_2_). These values were calculated as: CxO_2_ = (1,34 x Hb x SxO_2_) + (0,0031 x PxO_2_). The oxygen delivery content index (mL/min/kg) was calculated as DO_2_I = CaO_2_ x (CI / 100) and the oxygen consumption index (mL/min/kg) as VO_2_I = (CaO_2_ – CvO_2_) x (CI / 100). The oxygen extraction ratio (%) was defined as O_2_ER = VO_2_I / DO_2_I. The alveolar-arterial oxygen difference (PAO_2_ – PaO_2_) (kPa) was calculated as ([{barometric pressure – PH_2_O} x F_i_O_2_] – [PaCO_2_ x R–1]) – PaO_2_, where R is the respiratory exchange ratio with a value of 0.8. This simplified formula was used as FiO_2_ (0.21) was low. Shunt fraction (%) was calculated using the Berggren formula: Qs/Qt = (CcO2 - CaO2)/(CcO2 – CvO2) × 100 [38]. The barometic pressure, which was used for these calculations, was the specified barometic pressure in Hannover (Germany, 55 m above sea level) at the time of measurement.

### Statistics

GraphPad Prism 8 (GraphPad Software, San Diego, USA) was used for all statistical calculations and graphical illustration. A *p*-value of < 0.05 with a confidence interval of 95% was considered significant.

Data are presented as mean ± standard deviation. Normal distribution was tested by the Shapiro-Wilk test and visual inspection of the QQ-plots. A two-way ANOVA for repeated measures was used for statistical evaluation of normally distributed data, followed by post-hoc Bonferroni test. A one-way-ANOVA for repeated data, followed by post-hoc Dunnett test, was used to analyse changes over time in relation to a baseline value. A two-way-ANOVA for repeated data, followed by post-hoc Tukey test, was used to analyse changes over time during anaesthesia. A non-parametric Wilcoxon sign rank test was used to analyse the recovery scale data.

## Data Availability

The datasets used/or analyzed during the current study are available from the corresponding author on reasonable request.

## Abbreviations

C⊽O_2_: Mixed-venous oxygen content
CaO_2_: Arterial oxygen content
CO: Cardiac output
CO_2_: Carbon dioxide
DO_2_: Oxygen delivery
DO_2_I: Oxygen delivery index
E_T_CO_2_: End-tidal carbon dioxide
F_i_O_2_: Fraction of inspired oxygen
HR: Heart rate
INT: Intubated
LiDCO: Lithiumdilution Cardiac output
MAP: Mean arterial blood pressure
MPADP: Mean pulmonal-arterial blood pressure
nINT: Non-intubated
NO: Nitric oxide
O_2_: Oxygen
O_2_ER: Oxygen extraction rate
P_(A-a)_O_2_: Alveolar-arterial oxygen difference
P_(a-ET)_O_2_: Arterial-end-tidal carbon dioxide difference
P⊽CO_2_: Mixed-venous carbon dioxide partial pressure
P⊽O_2_: Mixed-venous oxygen partial pressure
PaCO_2_: Arterial carbon dioxide partial pressure
PAO_2_: Alveolar oxygen partial pressure
PaO_2_: Arterial oxygen partial pressure
ppb: Parts per billion
PvCO_2_: Venous carbon dioxide partial pressure
PvO_2_: Venous oxygen partial pressure
Q_s_/Q_t_: Shunt fraction
RR: Respiratory rate
S⊽O_2_: Mixed-venous oxygen saturation
SaO_2_: Arterial oxygen saturation
SvO_2_: Venous oxygen saturation
TIVA: Total intravenous anaesthesia
V_A_: Alveolar ventilation
V_A_/Q: Ventilation-perfusion ratio
VO_2_: Oxygen uptake
VO_2_I: Oxygen uptake index
